# MYB Transcription Factors Negatively Regulate *StL3OH* in Salt Stress Response of *Schizonepeta tenuifolia*

**DOI:** 10.3390/plants15101469

**Published:** 2026-05-12

**Authors:** Jingjie Dang, Maoqi Pan, Mengru Sang, Dishuai Li, Mingqiu Shan, Chanchan Liu, Qinan Wu

**Affiliations:** 1 National Key Laboratory on Technologies for Chinese Medicine Pharmaceutical Process Control and Intelligent Manufacture, Nanjing University of Chinese Medicine, Nanjing 210023, China; jingjie.dang@njucm.edu.cn (J.D.); pan12@njucm.edu.cn (M.P.); sang7923@163.com (M.S.); 20210651@njucm.edu.cn (D.L.); shanmingqiu@njucm.edu.cn (M.S.); 2 Jiangsu Collaborative Innovation Center of Chinese Medicinal Resources Industrialization, Nanjing University of Chinese Medicine, Nanjing 210023, China; 3 School of Pharmacy, Nanjing University of Chinese Medicine, Nanjing 210023, China

**Keywords:** *Schizonepeta tenuifolia* (Benth.) Briq., salt stress, MYB transcription factors, *StL3OH*, monoterpenoid biosynthesis

## Abstract

Salt stress affects the growth, quality, and secondary metabolism of medicinal plants, but its effects on essential oil biosynthesis in *Schizonepeta tenuifolia* remain unclear. In this study, a salt stress model was established for *Schizonepeta tenuifolia* (Benth.) Briq. to investigate changes in growth, monoterpenoid accumulation, non-targeted metabolism, and transcriptional profiles. To further clarify the regulatory mechanism of monoterpenoids biosynthesis, the MYB family members were identified at the genome-wide level, and candidate regulators were screened based on the expression patterns and promoter features of *StL3OH* (limonene -3-hydroxylase). Among them, *StMYB71* and *StMYB8774* were identified as candidate regulators of monoterpene biosynthesis. Functional analysis indicated that both transcription factors negatively regulated *StL3OH* expression by binding to the MYBHv1 cis-element in its promoter. These findings improve our understanding of the salt stress response and transcriptional regulation of monoterpene biosynthesis in *S. tenuifolia* and provide a basis for the future improvement of Schizonepetae Herba quality and salt tolerance.

## 1. Introduction

*Schizonepeta tenuifolia* (Benth.) Briq. belongs to the Lamiaceae family and is widely used in traditional Chinese medicine because of its antipyretic, antioxidant, anti-inflammatory, and antitumor activities [1]. Monoterpenoids, the major constituents of volatile oil from *S. tenuifolia*, are considered the principal bioactive compounds of *S. tenuifolia*. Therefore, elucidating the biosynthetic and regulatory mechanisms of monoterpenoids in *S. tenuifolia* is important for understanding quality formation in traditional Chinese medicine and for breeding high-quality varieties. Among them, (−)-pulegone is one of the predominant monoterpenoids and serves as an important quality evaluation indicator of Schizonepetae Herba. Based on genomic data, we previously elucidated the (−)-pulegone biosynthetic pathway in *S. tenuifolia* through in vitro and vivo assays [2]. StL3OH is the first structural modification enzyme in the pulegone biosynthetic pathway and could determine substituent positions in the final products [3]. In vitro enzyme assays showed that StL3OH mainly catalyzes the conversion of (+)-limonene to (+)-*cis*-isopiperitenol. Carvone was detected as a by-product of the StL3OH-catalyzed reaction in vitro and exhibited a substitution pattern different from that of (+)-*cis*-isopiperitenol, suggesting that StL3OH can generate products with different substitution positions [2]. However, products with alternative substitution patterns catalyzed by StL3OH have not been detected in the essential oils of *S. tenuifolia*. Elucidating the regulatory mechanism of StL3OH will therefore improve our understanding of terpenoid biosynthesis in *S. tenuifolia*.

Abiotic stress plays an important role in plant growth and the accumulation of secondary metabolites [4]. Previous studies have investigated the effects of abiotic stress on the quality and physiology of *S. tenuifolia* [5,6]. Salinity significantly affects trichome density, antioxidant capacity, essential oil content, and overall plant growth, indicating that salt stress has a profound impact on the quality of traditional Chinese medicines. In response to salt stress, plants undergo extensive physiological and metabolic reprogramming, which in turn influences the biosynthesis and accumulation of secondary metabolites [7]. Transcription factors play crucial roles in regulating these processes. Among them, the MYB family represents one of the largest classes involved in plant growth and secondary metabolism. In particular, the R2R3-MYB subfamily serves as a key regulatory group that can either positively or negatively modulate secondary metabolite biosynthesis [8,9]. Previous studies showed that StMIXTA, a member of subgroup 9 of the R2R3-MYB family, is involved in glandular trichome development and monoterpenoid biosynthesis [10]. In addition, R2R3-MYB transcription factors can interact with key sensors involved in salinity responses and thereby regulate plant physiology [11]. Thus, investigating MYB transcription factors may help connect changes in primary and secondary metabolism and further elucidate the mechanisms underlying quality formation and salt responses in traditional Chinese medicine.

In this study, we established a salt stress model for *S. tenuifolia* to characterize its responses to salinity. We further analyzed the changes in growth traits and physiological indices under salt stress. Malondialdehyde (MDA) content and peroxidase (POD) activity changed significantly, and plant growth was progressively inhibited with increasing salt concentration. Quality-related indicators of *S. tenuifolia* increased, as reflected by increased (−)-pulegone content and glandular trichome density. Non-targeted metabolomic analysis showed that lipids represented the largest class of differential metabolites, indicating their broad involvement in the salt response of *S. tenuifolia*. Using genomic and transcriptomic data, we identified members of the MYB family in *S. tenuifolia*. Co-expression analysis between MYB genes and *StL3OH* enabled the identification of candidate MYB regulators that may control monoterpenoid biosynthesis through StL3OH. Electrophoretic mobility shift assay (EMSA) and yeast one-hybrid (Y1H) assays demonstrated that StMYB71 protein and StMYB8774 protein bind to the MYB-binding site in the *StL3OH* promoter. Virus-induced gene silencing further indicated that these MYB transcription factors regulate monoterpenoid biosynthesis through their interaction with the *StL3OH* promoter. Collectively, these findings provide a theoretical basis for studies on the geo-authenticity of Schizonepetae Herba and improve our understanding of the mechanisms underlying quality formation in traditional Chinese medicine.

## 2. Materials and Methods

### 2.1. Plant Material and Salt Stress Treatment

The seeds of *S. tenuifolia* (Benth.) Briq. were collected from Hebei, China, and grown in a greenhouse at Nanjing University of Chinese Medicine, Jiangsu, China. The plant was grown under 27 °C and 70–75% humidity [12]. The *S. tenuifolia* plants were allowed to grow naturally before salt stress was applied [13]. Plant materials with four to five pairs of real leaves were used for the stress assays. Concentrations of 0 mM/L (control group, watering), 10 mM/L NaCl (low group), 50 mM/L NaCl (medium group), and 100 mM/L NaCl (high group) were applied to each group for 30 days. There were 40 to 50 plants of *S. tenuifolia* in each group. Three days before the stress treatment began, 50 mL of salt solution was applied daily. After that, the salt solution was applied at intervals of 1 to 2 days. The stress treatment was carried out at a rate of 50 mL per plant every three days. Leaves, stems, and roots from the control, medium-salt, and high-salt treatment groups were collected, briefly washed with sterile water, immediately flash-frozen in liquid nitrogen and stored at −80 °C until RNA extraction for RNA-seq analysis.

### 2.2. Trichome Density and Non-Glandular Length Measurement

Trichome density was assessed following a previously published method [3]. Briefly, trichome numbers and the length of non-glandular trichomes were automatically quantified using Axio Vision 4.7 software (Carl Zeiss Ltd., Hertfordshire, UK). The mean trichome density was calculated based on measurements taken across the entire leaf surface.

### 2.3. Determination of Physiological and Biochemical Parameters

Leaf samples (0.1 g) of *S. tenuifolia* were accurately weighed and homogenized in 2 mL PBS buffer with 2 stainless steel beads (5 mm diameter) using a tissue grinder at 60 Hz for 60 s. The homogenate was then centrifuged at 12,000 rpm for 10 min at room temperature, and the supernatant was collected for subsequent analysis.

Soluble protein content was determined using a BCA Protein Assay Kit (KeyGEN BioTECH, Nanjing, China) according to the manufacturer’s instructions, and quantified based on a standard curve.

The activities of peroxidase (POD), superoxide dismutase (SOD), and catalase (CAT) were measured using the same protein extract as the sample source, following the protocols provided by the assay kit manufacturer (KeyGene Biotech, China).

Malondialdehyde (MDA) content was determined according to the manufacturer’s instructions using a commercial assay kit, based on the thiobarbituric acid (TBA) method.

### 2.4. Volatile Oils Analysis

One gram of fresh leaves was transferred into 2 mL hexane containing 30 ng/mL camphor and essential oil was extracted by grinding [14]. Each sample contained three repeats for GC and GC-MS analysis.

Gas chromatography-mass spectrometry (GC-MS) was performed using a 6890N GC interfaced with 5973 inert MS instrument (Agilent Technologies, Santa Clara, CA, USA). Compounds were separated on an HP-5MS capillary column (30 m × 250 μm × 0.25 μm). The oven temperature program was as follows: it was kept at 50 °C for 3 min, then raised to 90 °C at 3 °C/min, then raised to 150 °C at 5 °C/min and finally raised to 220 °C at 10 °C/min for 5 min. The injection volume was 1.0 μL in splitless mode. The mass spectrometer was operated in electron impact (EI) mode at 70 eV. The ion source temperature was set at 230 °C and the quadrupole temperature was set at 150 °C. Mass spectra were recorded in full scan mode (*m*/*z* 40–500). Metabolites were identified based on chromatographic and spectral comparisons against the NIST 05 library. The standard of n-alkanes was diluted by n-hexane and the retention index was calculated as previously described [12].

Gas chromatography was performed using the 8860 GC (Agilent Technologies, Santa Clara, CA, USA) and HP-5 capillary column (30 m × 0.32 mm × 0.25 μm). The oven temperature program was the same as the GC-MS methods. The injection volume was 1.0 μL and splitless. The temperature of the injection port was 220 °C, and the FID detector temperature was 250 °C.

### 2.5. Extraction and Analysis of Non-Target Metabolism

One gram of powder from different organs from *S. tenuifolia* under salt stress was extracted with 10 mL of 80% methanol in a 50 mL flask. After a 10 min soaking period, ultrasonic extraction was carried out for 30 min. The mixture was centrifuged at 13,000 rpm for 10 min, and the supernatant was were obtained after filtration with a 0.22 μm membrane.

Metabolomic profiling was performed using a high-resolution AB SCIEX Triple TOF™ 5600 mass spectrometer equipped with a DuoSpray™ ion source operating in electrospray ionization (ESI) mode. Chromatographic separation was achieved on a Waters XBridge™ C18 column (5 μm, 4.6 × 250 mm, Milford, MA, USA) maintained at 30 °C. The LC system consisted of an LC-20ADXR pump (Shimadzu, Kyoto, Japan) and a SIL-20AC autosampler (Shimadzu, Kyoto, Japan). The flow rate was set at 1.0 mL/min with an injection volume of 10 μL.

The mobile phase comprised solvent A (0.1% formic acid in water) and solvent B (acetonitrile), using a programmed gradient elution: 0–30 min, 5–35% B; 30–35 min, 35–70% B; 35–40 min, 70–95% B; 40–45 min, 95–5% B; and 45–50 min, 5% B for re-equilibration.

Mass spectrometric detection was conducted in both positive and negative ionization modes. The ion source temperature was maintained at 600 °C, with an ion spray voltage of −4500 V and a declustering potential of −100 V. Collision energy was set to −10 V. The nebulizer gas (GS1) and auxiliary gas (GS2) were both maintained at 60 psi, while the curtain gas was set at 40 psi. MS and MS/MS spectra were acquired over an *m*/*z* range of 50–2000. Dynamic background subtraction (DBS) was applied during data acquisition.

Raw data were processed using Analyst^®^ TF 1.6 (AB SCIEX, Framingham, MA, USA) and MS-DIAL (version 4.80). For feature extraction, MS1 and MS2 mass tolerances were set to 0.01 Da and 0.025 Da, respectively, with a retention time window of 100 min and an *m*/*z* detection range of 50–2000 Da. The minimum peak height threshold was defined as 80 amplitude units, and the mass slice width was 0.1 Da. Peak smoothing was performed using a linear weighted moving average method with a sigma value of 0.5. MS/MS signals below the abundance threshold were excluded.

Metabolite annotation was achieved by matching experimental spectra against the MassBank of North America database (.msp format), supplemented by in-house spectral libraries. Identification criteria included MS1 and MS2 tolerances of 0.01 and 0.05 Da, respectively. Retention time alignment was corrected within 0.5 min using an MS1 tolerance of 0.015 Da. The resulting annotation table was exported in TXT format and manually curated to eliminate potential false positives [15].

### 2.6. RNA-Seq

#### 2.6.1. RNA Extraction, Library Construction, and Sequencing

Samples for RNA-seq were collected separately from *S. tenuifolia*. Total RNA was extracted using Trizol reagent (Thermofisher, Waltham, MA, USA, 15596018) following the manufacturer’s procedure. Bioanalyzer 2100 and RNA 6000 Nano LabChip Kit (Agilent, Santa Clara, CA, USA, 5067-1511) were used for analyzing the quality and purity of *S. tenuifolia.* RNA samples were sequenced with Illumina NovaseqTM 6000 sequence platform by LC Bio Technology Co., Ltd. (Hangzhou, China).

#### 2.6.2. Alignment with Genome and Different Expression Genes Analysis

All samples were aligned to the genome using HISAT2 (https://daehwankimlab.github.io/hisat2/, version: hisat2.2.1, accessed on 5 October 2024) package, which initially removed a portion of threads based on quality information accompanying each read and then mapped the reads to the genome published previously [2]. The mapped reads of each sample were assembled using StringTie (http://ccb.jhu.edu/software/stringtie/, version: 2.1.6, accessed on 5 October 2024) with default parameters.

Differentially expressed genes (DEGs) analysis was performed by DESeq 2 between two different groups. The genes with the parameter of a false discovery rate (FDR) below 0.05 and an absolute fold change of ≥2 were considered differentially expressed genes. Differentially expressed genes were then subjected to enrichment analysis of GO functions and KEGG pathways. GO enrichment analysis provided all GO terms that significantly enriched in DEGs compared to the genome background. Firstly, all DEGs were mapped to GO terms in the Gene Ontology database (http://www.geneontology.org/), gene numbers were calculated for every term, and significantly enriched GO terms in DEGs compared to the genome background were defined using a hypergeometric test [16]. KEGG is the major public pathway-related database. Pathway enrichment analysis identified significantly enriched metabolic pathways or signal transduction pathways in DEGs compared with the whole genome background [17].

### 2.7. Quantitative Real-Time PCR Analysis

The samples collected in Section 2.1 and leaves of *S. tenuifolia* from VIGS were used for quantitative real-time PCR analysis. Total RNA was extracted using a FastPure Plant Total RNA Isolation Kit (Polysaccharides & Polyphenolics–rich; Vazyme, Nanjing, China). The cDNA was synthesized with 50 ng of total RNA by the HiScript III 1st Strand cDNA Synthesis Kit (+gDNA wiper) (Vazyme, Nanjing, China). The Hieff^®^ qPCR SYBR Green Master Mix was used to conduct the qPCR reaction according to the manufacturer’s procedure. The *S. tenuifolia* β-actin gene was used as a control to normalize the relative expression levels of the target genes and the relative expression of target genes was calculated by 2^−ΔΔCT^. Three biological replicates were performed in all experiments. The primers used for qRT-PCR are listed in Table S1.

### 2.8. The Bioinformation Analysis of the MYB Family Members and Coexpression Analysis

The MYB-related family member sequence of *Arabidopsis thaliana* was downloaded from TAIR (https://www.arabidopsis.org/). Two-way BLAST was used to identify the member of the MYB-related family members in *S. tenuifolia*. MEME (http://meme-suite.org/tools/meme, accessed on 10 October 2025) and Batch-CD online website (https://www.ncbi.nlm.nih.gov/Structure/bwrpsb/bwrpsb.cgi, accessed on 10 October 2025) was used to analyze the motif and conversed domain of the MYB-related family. The protein sequence of MYB-related members in *Arabidopsis thaliana* and *S. tenuifolia* was aligned by muscle v5.1, and maximum likelihood phylogenetic tree was constructed by iqtree v2.2.6 and visualized by the iTOL online website (https://itol.embl.de/itol.cgi, accessed on 10 October 2025). The TBtools v2.042 was used to predict the distribution of exon and intron and gene location and visualization of analysis results [18]. The published transcriptomes from *S. tenuifolia* were downloaded from NCBI. Gene co-expression analysis was performed on the transcription data by using *StL3OH* as bait gene.

### 2.9. Y1H Assay

The promoters of *StL3OH28415* were synthesis by Sangon (Shanghai, China) and subcloned into the pNC-AbAi vector by nimble cloning according to the manufacturer’s procedure to get the pNC-AbAi-StL3OHpro vector. The CDS of candidate genes were subcloned into pNC-GADT7 vector by nimble cloning to obtain the vectors of pNC-AD-MYB71 and pNC-AD-MYB8774. The reconstructed vectors were sequenced by Sangon (Shanghai, China). The Y1H assay was conducted using YIH Gold competent cells, which were purchased from the WeidiBiotech (Shanghai, China). The assays were performed as previously described [19]. The vector of pNC-AbAi-StL3OHpro was digested by BstBI and the linearized vector was transformed into YIH Gold competent cell according to the manuscript for the self-activation monitoring and test of Aureobasidin A inhibitory concentration. The vectors of pGADT7-MYB71 and pGADT7-MYB8774 were transformed into Y1H Gold competent cells containing the pNC-AbAi-StL3OHpro vector. The pGADT7 were also co-transformed into the same yeast competent cells as the negative control. The transformation products were incubated on SD/-Trp/-Ura plates, and positive clonnies were transferred to SD/-Trp/-Ura plates containing 600 ng/mL Aureobasidin A. The primers used for the Y1H assays are listed in Table S2.

### 2.10. Electrophoretic Mobility Shift Assay

For protein expression and purification, the specific primers of StMYB8774 and StMYB71 were designed and synthesized by Sangon (Shanghai, China) for the construction of the recombined vector of pGEX-6p-1, and the constructs were transferred into *Escherichia coli* BL21 competent by heat shocking. The single colony was inoculated into an LB broth containing 50 mg/L ampicillin and incubated at 37 °C to reach an OD600 of approximately 0.8. The culture was supplemented with 1 mM isopropyl-b-D-thiogalactoside and incubated at 16 °C for 24 h to induce the protein expression. The cells were harvested by centrifugation (15 min at 4 °C and 5000 rpm) and were resuspended in the buffer containing 10 mM Tris-HCl, 200 mM NaCl, and 5% glycerol. Then, the cells were broken by ultrasonication. The crude protein was obtained by centrifugation (30 min at 4 °C and 12,000 rpm) and purified by Ni-NTA gravity column according to the manufacturer’s instructions to obtain the protein of GST-MYB71 and GST-MYB8774.

An EMSA detected kit was purchased from the Beyotime Biotechnology (Shanghai, China). A total of 0.5 μg of recombined proteins of GST-MYB71 and GST-MYB8774 was used for the assays. The MYB-binding and MYB-competitor probes were synthesized by Sangon (Shanghai, China). The assays were performed according to the manufacturer’s procedure. The primers and the probes used in the assays are shown in Table S3.

### 2.11. Virus-Induced Gene Silence

Virus-induced gene silencing (VIGS) was performed as previously described [2] with minor modifications. The VIGS system used in this study was based on the tobacco rattle virus (TRV) system, which consisted of the helper vector TRV1 and the gene-silencing vector TRV2.

The gene-specific fragments of *StMYB71* and *StMYB8774* were amplified using 2 × Phanta Flash Master Mix (Vazyme, Nanjing, China) and cloned into the TRV2 vector via homologous recombination. The recombinant TRV2 constructs were verified by sequencing (Sangon Biotech, Shanghai, China). TRV1 was used as the helper plasmid to support viral replication and systemic infection. Primer sequences used for vector construction are listed in Table S4.

The verified TRV1 and TRV2 constructs were separately transformed into *Agrobacterium tumefaciens* strain GV3101. Positive colonies were cultured and resuspended in infiltration buffer containing 10 mM MgCl_2_, 10 mM MES, and 200 μM acetosyringone (pH 5.6). The TRV1 and TRV2 cultures were mixed at a 1:1 ratio and incubated at room temperature for 2–3 h before infiltration.

Ten-day-old *S*. *tenuifolia* seedlings were used for agroinfiltration. Plants infiltrated with TRV2 empty vector served as the negative control. After infiltration, plants were maintained in the dark at 25 °C for 24 h and then transferred to normal growth conditions. Twenty days after infiltration, leaf samples were collected for subsequent analysis. Silencing efficiency was evaluated by quantitative real-time PCR (qRT-PCR) analysis of target gene expression levels compared with the control group.

To analyze changes in volatile metabolites, 0.1 g of fresh leaves was ground in hexane containing 30 ng/mL camphor as an internal standard, and essential oil components were analyzed by GC analysis. In addition, the reliability of this system in our laboratory has been previously validated using the phytoene desaturase (PDS) gene, which produced a visible photobleaching phenotype, confirming system efficiency.

### 2.12. Statistical Analysis

The statistical analysis of significant differences between different groups was tested using one-way analysis of variance and a nonparametric test with IBM SPSS statistics 26 software (SPSS, USA). Different lowercase letters showed statistically significant at *p* < 0.05.

## 3. Results

### 3.1. Salt Imposes a Major Constraint on S. tenuifolia Growth

**Figure 1:**
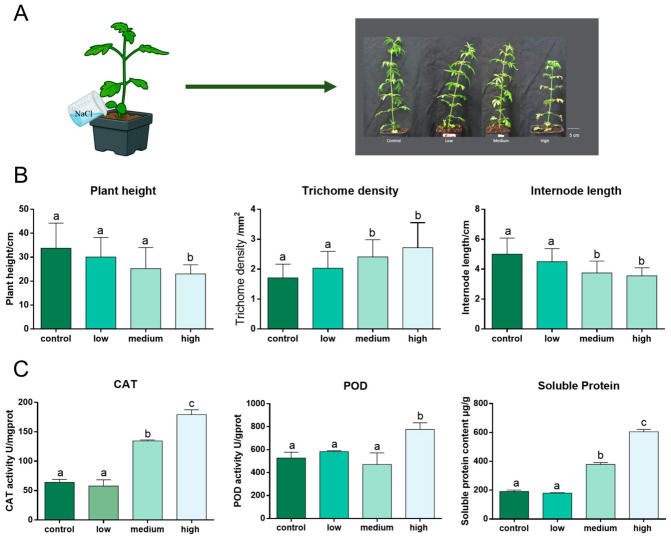
Phenotypic and physiological responses of *S. tenuifolia* under salt stress. (**A**) Phenotypic changes of *S. tenuifolia* under different salt treatments; (**B**) Effects of salt stress on plant height, trichome density and internode length; (**C**) Changes in CAT activity, POD activity and soluble protein content under salt stress. Different lowercase letters indicate significant differences among treatments (*p* < 0.05).

Salt stress can inhibit plant growth and cause cellular damage [20]. To investigate the responses of *S. tenuifolia* to salt stress, one-month-old seedlings were selected as experimental materials (Figure 1A). A salt stress model was established by irrigating plants with salt solutions of different concentrations for 1 month. As shown in Figure 1B, plant height decreased as salt concentration increased, with the strongest inhibition observed in the medium- and high-salinity groups. A significant difference in plant height was observed between the high-salinity group and the other groups. During the treatment period, growth rates declined under salt stress (Figure S1B). Internode length increased gradually during development (Figure S1C), but was also significantly affected by salt stress, particularly in the medium- and high-salinity groups. Although plant height did not differ significantly between the control and medium-salinity groups, internode length showed a significant difference (Figure 1B). In addition, the leaves of *S. tenuifolia* were visibly damaged under salt stress, and the severity of injury increased with increasing salt concentration (Figure S1A). These results indicated that elevated salinity inhibited the growth of *S. tenuifolia*. To evaluate physiological responses to salt stress, superoxide dismutase (SOD), catalase (CAT), peroxidase (POD), malondialdehyde (MDA), soluble protein, and proline were measured. As shown in Figure 1C, CAT activity increased significantly in the medium- and high-salinity groups, with a significant difference also observed between these two groups. The trend in soluble protein content was consistent with that of CAT activity during salt treatment. POD activity was significantly higher in the high-salinity group than in the control, low-, and medium-salinity groups. As shown in Figure S2, SOD activity did not differ significantly among the groups, whereas MDA content increased significantly in the medium- and high-salinity groups, and proline content increased significantly in the high-salinity group. These results suggest that CAT activity, POD activity, and soluble protein accumulation were the physiological indices most responsive to salt stress.

**Figure 2:**
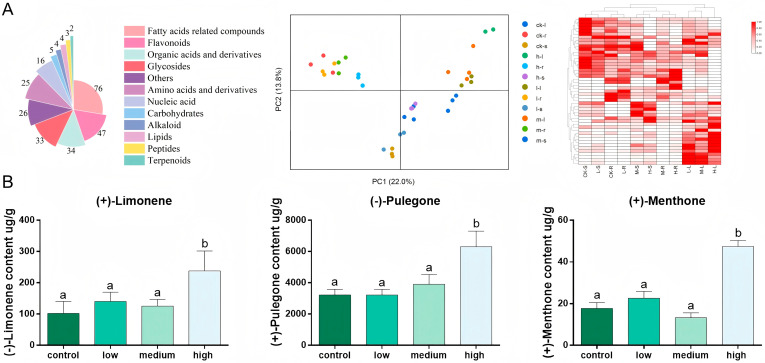
The analysis of metabolites from *S. tenuifolia* under salt stress. (**A**) The species of metabolite and PCA analysis of non-target metabolism and the heatmap of lipids peak areas from non-target metabolomics; (**B**) the content of (−)-limonene, (+)-pulegone, and (+)-menthone from *S. tenuifolia* under salt stress by GC. Different lower case letters mean statistically significant at *p* < 0.05.

In this study, a total of 261 compounds were identified, including 89 lipids and their derivatives, 33 fatty acids and their derivatives, 30 organic acids and their derivatives, 28 terpenoids and their derivatives, 20 flavonoids, 17 nucleic acids and their derivatives, 11 amino acids and their derivatives, 11 glycosides, 10 carbohydrates, 6 phenylpropanoids, 4 alkaloids, and 2 lignans (Figure 2A). Plant lipids are major structural components of cellular and organellar membranes and play essential roles in plant responses to both biotic and abiotic stresses. For example, unsaturated fatty acids contribute to resistance against pests and pathogens, whereas fatty acid derivatives help maintain osmotic balance and reduce cellular damage under abiotic stress conditions [21,22]. The lipid heatmap showed that most lipids accumulated at higher levels in leaves than in roots. Moreover, lipid levels generally increased with increasing salinity, further indicating that lipid metabolism is broadly involved in the salt stress response of *S. tenuifolia*.

### 3.2. Salinity Positively Regulates the Content of Essential Oil from S. tenuifolia

Glandular trichomes originate from plant epidermal cells and function in defense and detoxification [23]. Both non-glandular hairs and glandular trichomes are characteristic epidermal structures of *S. tenuifolia* [24]. Interestingly, we found that non-glandular hair length decreased during plant development, a phenomenon that has not been reported previously (Figure S3E). However, no significant differences in non-glandular hair length were observed among the treatment groups. Glandular trichomes are considered the main sites of essential oil accumulation [25]. During plant development, trichome diameter gradually increased (Figure S3D), indicating the accumulation of volatile constituents in the leaves of *S. tenuifolia*. Salinity did not significantly affect trichome diameter or non-glandular hair length (Figure S3A,B). In contrast, trichome density increased with increasing salt concentration. As shown in Figure 1B, trichome density was significantly higher in the medium- and high-salinity groups than in the control group. These results suggest that salinity may improve the quality-related traits of *S. tenuifolia* by increasing glandular trichome density.

GC–MS and GC were used to evaluate the quality of *S. tenuifolia*. A total of 19 compounds were identified by comparison with the NIST library and are listed in Table 1. The total ion chromatograms and monoterpenoid contents of different organs of *S. tenuifolia* are shown in Figure S4. Monoterpenoids were the major bioactive constituents of *S. tenuifolia*. GC–MS analysis showed no obvious differences in the qualitative composition of essential oils among the treatment groups. We further quantified (+)-limonene, (−)-pulegone, and (+)-menthone in *S. tenuifolia* by GC, and the analytical method was validated previously in our laboratory [26]. Salinity significantly affected the contents of (+)-limonene and (−)-pulegone, particularly in the medium- and high-salinity groups (Figure 2B). With prolonged NaCl treatment, the content of (+)-limonene decreased, whereas that of (−)-pulegone increased. The content of (+)-menthone increased significantly in the high-salinity group, whereas no significant changes were observed in the other groups. These results suggest that limonene was progressively converted into pulegone during plant growth. Pulegone content is used as a quality indicator for this herb in the Chinese Pharmacopoeia.

The biosynthetic pathway of menthone in *S. tenuifolia* has been elucidated [2,3,27,28]. Because the most pronounced effects of salt stress on plant growth and quality were observed in the medium- and high-salinity groups, different organs from the control, medium-, and high-salinity groups were collected for RNA-seq analysis. Based on the transcriptomic data, an expression heatmap was generated using TBtools (Supplementary Figure S7). *StLS*, *StIPD*, and *StIPR-2* were highly expressed in leaves. *StL3OH* belongs to the cytochrome P450 family and is a member of the CYP71D subfamily. Cytochrome P450 enzymes play important roles in plant growth and development, which may partly explain the dynamic expression pattern of *StL3OH* [29]. Based on the transcriptome expression matrix, self-organizing map (SOM) analysis was used to identify gene expression clusters. A total of 25,211 genes were divided into six classes, among which Class 6 contained the largest number of genes (Figure S5A). *StLS*, *StL3OH*, *StIPD*, and *StIPR*, which participate in pulegone biosynthesis, were assigned to the same class, indicating coordinated transcriptional responses to salinity. Overall, these results indicate that although salt stress inhibited plant growth, it promoted the accumulation of quality-related metabolites in *S. tenuifolia*.

### 3.3. The MYB Family Regulates the Content of Essential Oil by Regulating the Expression of StL3OH

**Figure 3:**
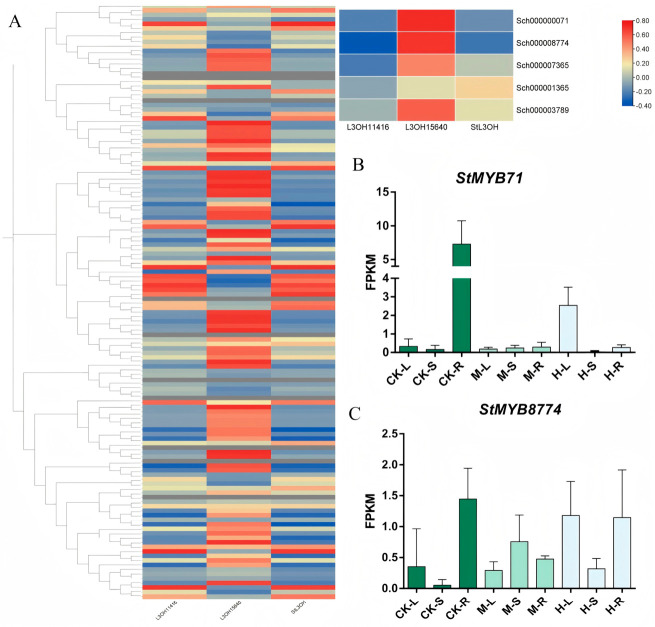
(**A**) The expression pattern of the MYB family and subgroup of candidate genes; (**B**) the FPKM (fragments per kilobase of transcript per million mapped reads) of candidate gene *StMYB71*; (**C**) the FPKM (fragments per kilobase of transcript per million mapped reads) of the candidate gene *StMYB8774*.

As shown in Figure 3A, PCA analysis of RNA-seq showed the transcription level of *S. tenuifolia* root is different from that of leaves and stem, which was consistent with the results of non-target metabolism. The differentially expressed genes between different organs were identified as grow-DEGs, while the differentially expressed genes were clarified as salt-response-DEGs. A total of 16,598 genes were identified as grow-DEGs. The results of KEGG enrichment find that the grow-DEGs were mainly focused on the sphingolipid metabolism, citrate cycle, and so on, indicating that the DEGs between different organs mainly concentrate on the plant growth and development and primary growth (Supplementary Figure S5). The result of GO enrichment showed similar results. Salt-response-DEGs in leaf and stem were enriched in secondary metabolism and biosynthesis in amino acids, while the salt-response-DEGs in root mainly focused on the metabolite biosynthesis involved in plant development. The enrichment result showed that protein and secondary metabolites can help the plant resist salt response. A lot of genes are involved not only in *S. tenuifolia* growth but also in its response to the salinity. As shown in Supplementary Figure S6A, a total of 200 genes are involved in plant growth and salinity response of leaf, stem, and root. The number of DEGs in both growth and salt response of leaf was 2211, and the enrichment analysis found that the genes were mostly enriched in metabolism of secondary metabolites. The 14,681 genes of growth and salt response in stem were also rich in metabolism of amino acids and secondary metabolites. There were 3018 DEGs involved in salt response of root and plant development, and they were mostly gathered in primary metabolites, such as glycan, lipids, and amino acids. Non-target metabolism showed lipids were crucial in the growth and stress defense of *S. tenuifolia*. They can be also involved in the regulation of secondary metabolism as signaling molecules.

MYB transcription factors can regulate POD activity and proline biosynthesis, thereby enhancing plant salt tolerance, and may play important roles in the salt stress response of *S. tenuifolia* [30]. Previous studies have also shown that MYB transcription factors were involved in the regulation of monoterpenoid biosynthesis and more broadly participate in the control of plant secondary metabolism [31]. To further investigate the regulatory roles of MYB transcription factors in *S. tenuifolia*, we performed a genome-wide analysis of the MYB family. Using the sequences of *Arabidopsis thaliana* R2R3-MYB proteins as queries, a total of 134 proteins were identified as the R2R3-MYB family members in *S. tenuifolia*. The results are shown in Figure S8 and Table S6. The coding sequence lengths ranged from 246 to 3321 bp, and the predicted proteins ranged from 82 to 1107 amino acids in length. The theoretical isoelectric points ranged from 3.72 to 10.03, and the molecular weights ranged from 8.94 to 124.08 kDa. MEME analysis showed that most R2R3-MYB proteins contained motif 5 and motif 3 (Figure S8B). Conserved domain analysis indicated that most R2R3-MYB members in *S. tenuifolia* contained domains belonging to the PLN03091 and PLN03212 superfamilies, consistent with previous reports [32]. Phylogenetic analysis of *S. tenuifolia* MYB proteins, together with those from *A. thaliana*, showed that these proteins were classified into 25 subgroups. Based on the genomic data, we further analyzed the chromosomal distribution and gene structures of the R2R3-MYB family using TBtools. The results showed that most members contained three exons and were distributed across HiC-Scaffold 1 to HiC-Scaffold 6. According to the classification system of the *A. thaliana* R2R3-MYB family, the *S. tenuifolia* R2R3-MYB proteins were assigned to 23 subgroups.

*StL3OH* is considered a key enzyme in monoterpenoid biosynthesis in *S. tenuifolia* [3]. Using *StL3OH* as the bait gene, we further performed co-expression analysis between *StL3OH* and the MYB family members in *S. tenuifolia*. The results showed that several MYB genes were positively correlated with *L3OH15640* expression, but negatively correlated with *StL3OH* and *L3OH11416* expression (Figure 3A). These findings suggest that the MYB family members may regulate monoterpenoid biosynthesis through coordinated regulation of L3OH-related genes.

### 3.4. MYB Family Members Directly Interact with StL3OH Through the MYBHv1-Binding Site

In vitro and in vivo enzyme assays indicated that *L3OH28415* is the major enzyme involved in pulegone biosynthesis and was therefore designated as *StL3OH*. The promoter sequence of *StL3OH* was obtained from the published genome of *S. tenuifolia* and analyzed using the PlantCARE database. The results showed that the *StL3OH* promoter contained a MYB-binding site that differed from those of other *CYP450* promoters. Based on the published genome data of *S. tenuifolia*, two MYB genes homologous to *MYBHv1* were identified. *Sch000000071* and *Sch000008774* were designated as *StMYB71* and *StMYB8774*, respectively. The expression patterns of *StMYB71* and *StMYB8774* are shown in Figure 3B,C. The results indicated that salt stress affected the expression of these candidate genes, both of which were highly expressed in the roots of the control group. After salt treatment, *StMYB71* expression increased in leaves, whereas *StMYB8774* expression was markedly upregulated in the high-salinity group.

**Figure 4:**
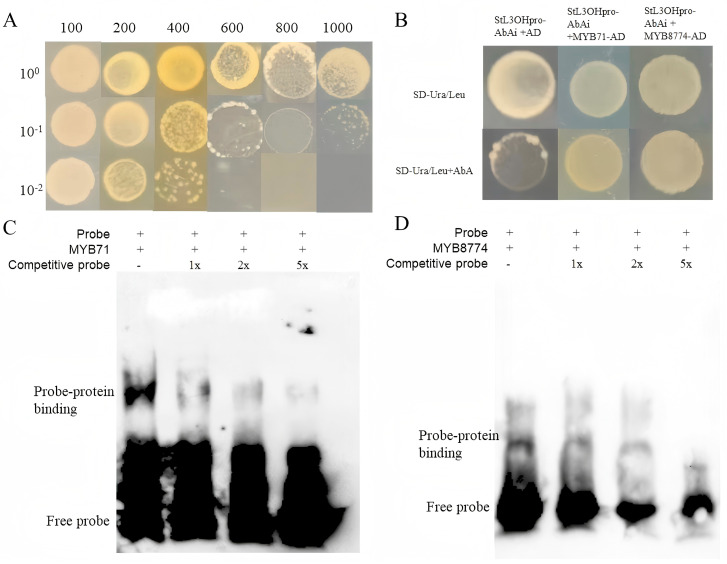
(**A**) The self-activation assays of StL3OHpro-AbAi; (**B**) Y1H assays of candidate MYB members and StL3OHpro-AbAi; (**C**) the EMSA assays of StMYB71 and the MYBHV1-binding site (the full-length gel is shown in Supplementary Figure S9); (**D**) the EMSA assays of StMYB8774 and the MYBHV1-binding site.

Self-activation analysis showed that the appropriate inhibitory concentration of Aureobasidin A was 600 ng/mL (Figure 4A). Yeast one-hybrid (Y1H) assays showed that *StMYB71* and *StMYB8774* directly bound to the *StL3OH* promoter (Figure 4B). Electrophoretic mobility shift assay (EMSA) further demonstrated that *StMYB71* and *StMYB8774* can bind to the MYBHv1 cis-element in the *StL3OH* promoter, suggesting their potential involvement in the regulation of *StL3OH* expression (Figure 4C,D).

**Figure 5:**
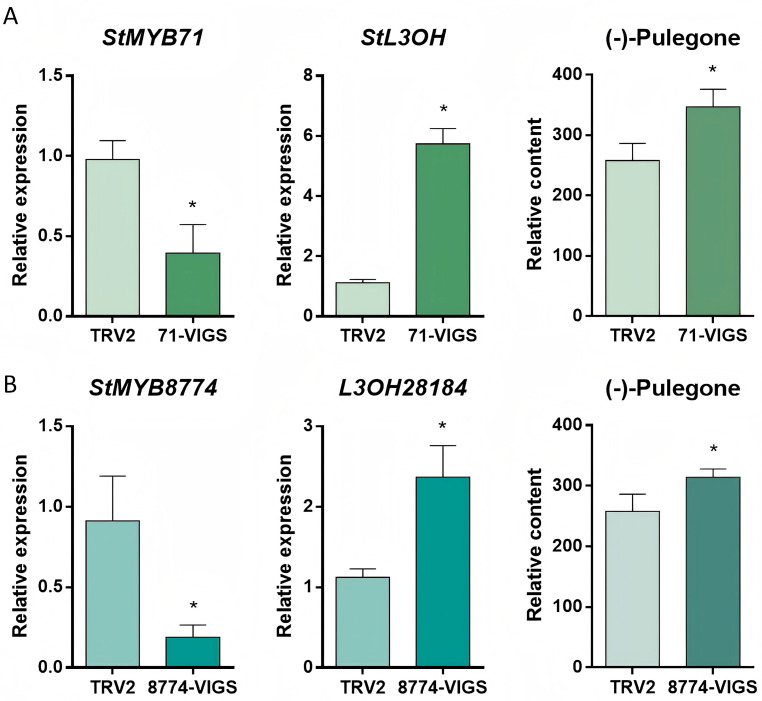
VIGS assays of *StMYB71* and *StMYB8774*. (**A**) The expression level of *StMYB71* and *StL3OH*, and the (−)-pulegone content after the silence of *StMYB71*; (**B**) the expression level of *StMYB8774* and *StL3OH*, and the (−)-pulegone content after the silence of *StMYB8774*. * means statistically significant at *p* < 0.05.

Virus-induced gene silencing (VIGS) assays were performed in *S. tenuifolia* seedlings. After infection, leaves were collected for RNA extraction and GC analysis. As shown in Figure 5, the expression levels of *StMYB71* and *StMYB8774* decreased by 50% and 75%, respectively. GC analysis showed that the relative contents of pulegone and limonene increased after *StMYB71* silencing. A similar trend in monoterpenoid accumulation was observed following *StMYB8774* silencing. qRT-PCR analysis showed that *StL3OH* expression increased by nearly sixfold when *StMYB71* was silenced, whereas inhibition of *StMYB8774* increased *StL3OH* transcription by nearly twofold. These results indicate that *StMYB71* and *StMYB8774* negatively regulate *StL3OH* expression by binding to the *MYBHv1* site in its promoter, thereby modulating monoterpenoid biosynthesis.

## 4. Discussion

In this study, we investigated the effects of salt stress on the quality and physiological responses of *S. tenuifolia*. Non-targeted metabolomic analysis indicated that lipids were extensively involved in the salt stress response of *S. tenuifolia*. Transcriptomic analysis further revealed salt stress-induced changes in gene expression, including altered expression patterns of enzymes involved in pulegone biosynthesis. Based on the genomic and transcriptomic data, the MYB family members in S. tenuifolia were systematically identified. Co-expression analysis suggested that specific MYB transcription factors may be involved in the regulation of pulegone biosynthesis. Promoter analysis further identified two MYB members that may regulate *StL3OH* transcription through direct interaction with its promoter, thereby affecting monoterpenoid biosynthesis. Y1H and EMSA were subsequently performed to verify the interactions between the candidate MYB transcription factors and the StL3OH promoter. In addition, virus-induced gene silencing (VIGS), together with gene expression and monoterpenoid content analyses, further confirmed the regulatory roles of these candidate MYB factors.

Abiotic stress affects plant growth and physiological status, including plant height, leaf area, chlorophyll content, stomatal conductance, and hormone biosynthesis [4]. Previous studies have shown that drought stress, exogenous hormone treatments, and salt stress alter the growth and physiological status of *S. tenuifolia* [33]. In the present study, salinity inhibited plant growth and internode elongation. Physiological analyses further showed that *S. tenuifolia* responded to salt stress through increased soluble protein and malondialdehyde (MDA) contents, as well as enhanced catalase (CAT) and peroxidase (POD) activities, indicating the activation of osmotic adjustment and antioxidant defense systems under salinity conditions.

Abiotic stress can also regulate the biosynthesis of secondary metabolites. For example, artemisinin and flavonoid biosynthesis in *Artemisia annua* is influenced by salinity, low temperature, and drought stress. The quality of traditional Chinese medicinal materials is closely associated with both the accumulation of secondary metabolites and the morphological characteristics of the medicinal organs. Therefore, trichome density and monoterpenoid content were used as indicators to evaluate the quality of *S. tenuifolia* under salt stress. GC and GC–MS analyses showed that salinity increased both trichome density and monoterpenoid content, suggesting that salt stress may improve the quality-related traits of *S. tenuifolia*. The increase in monoterpenoid accumulation under salt stress may be closely associated with the observed phenotypic and developmental changes in *S. tenuifolia*. Salinity inhibited plant growth and internode elongation, suggesting that metabolic resources may be redirected from primary growth processes toward stress adaptation and secondary metabolism. In addition, the increased trichome density observed under salt stress may provide more specialized structures for monoterpenoid biosynthesis and storage, thereby contributing directly to the enhanced accumulation of volatile compounds. These results suggest that salt-induced morphological adaptations are closely linked to the regulation of monoterpenoid production in *S. tenuifolia*.

To determine whether the increase in monoterpenoid content was simply caused by reduced biomass under salt stress, plants subjected to salt treatment were harvested and dried according to the procedures described in the Chinese Pharmacopoeia, and essential oils were subsequently extracted and analyzed by GC and GC–MS. Monoterpenoid content increased in all salt-treatment groups, with the greatest increase observed under moderate salinity. Interestingly, although high salinity also promoted monoterpenoid accumulation, severe leaf damage was observed under this condition. In contrast, the moderate-salinity treatment resulted in relatively less tissue damage while maintaining higher monoterpenoid accumulation, suggesting that moderate salt stress may optimize the balance between stress adaptation and secondary metabolite biosynthesis. These findings indicate that appropriate salinity treatment may improve the quality-related traits of Schizonepetae Herba (Figure S9).

Transcription factors regulate target genes either by directly binding to cis-regulatory elements or by interacting with other transcription factors. Several transcription factor families, including WRKY, AP2/ERF, bZIP, bHLH, and MYB, play important roles in plant growth, stress responses, and the biosynthesis of secondary metabolites [34]. In the present study, co-expression analysis suggested that MYB transcription factors may be involved in the regulation of monoterpenoid biosynthesis in *S. tenuifolia*. Based on promoter analysis of *StL3OH*, two MYB genes were identified as candidate regulators. Yeast one-hybrid (Y1H) and electrophoretic mobility shift assay (EMSA) further confirmed that these candidate MYB transcription factors directly interacted with the *StL3OH* promoter. In addition, virus-induced gene silencing (VIGS) assays demonstrated that these MYB genes negatively regulate monoterpenoid biosynthesis in *S. tenuifolia* by repressing *StL3OH* transcription. These findings suggest that the candidate MYB transcription factors may contribute to the coordination of salt-responsive physiological changes and essential oil biosynthesis. However, because a stable genetic transformation system is not yet available for *S. tenuifolia*, the precise roles of these MYB genes in salt stress responses require further investigation.

## 5. Conclusions

**Figure 6:**
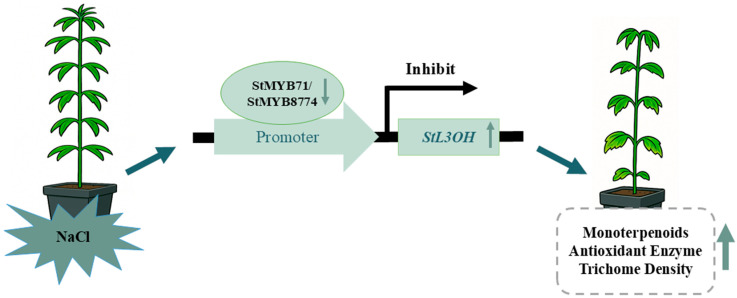
The hypothesis model of salt response and metabolite regulation from *S. tenuifolia* under salt stress.

In conclusion, this study systematically characterized the morphological, physiological, and transcriptional changes of *S. tenuifolia* under salt stress and further elucidated the regulatory mechanism underlying essential oil biosynthesis and salt stress responses (Figure 6). Based on genomic and transcriptomic data, the MYB gene family in *S. tenuifolia* was identified at the whole-genome level and subjected to comprehensive bioinformatic analysis. Two candidate MYB genes were subsequently selected, and their roles in monoterpenoid biosynthesis were experimentally verified. Elucidation of the functions of these MYB transcription factors improves our understanding of the biosynthetic and regulatory mechanisms of monoterpenoids in plants under salt stress. Collectively, these findings provide new insights into the molecular basis underlying the quality formation of traditional Chinese medicine.

## Figures and Tables

**Table 1:** Main components of volatile oil in *Schizonepeta tenuifolia* (Benth.) Briq. under salt stress.

ID	Compound	CAS	Formula	Retention Time	Retention Index
1	p-Xylene	106-42-3	C8H10	5.64	926.0457774
2	Benzeneethanol	52059-32-4	C10H14	6.37	1233.333333
3	Nonane	111-84-2	C10H20	6.68	1961.674009
4	β-Pinene	127-91-3	C10H16	10.18	1411.40625
5	(+)-Limonene	5989-27-5	C10H16	11.74	1025.569761
6	Octane	3221-61-2	C9H20	13.14	1105.5505
7	trans-2-decalone	26532-25-2	C10H16O	15.1	2798.319328
8	(+)-Menthone	3391-87-5	C10H18O	17.37	1192.574032
9	(−)-Isopulegone	29606-79-9	C10H16O	18.31	1336.812749
10	(−)-Pulegone	3391-90-0	C10H16O	20.72	1250.195567
11	Decane	192823-15-7	C14H30	22.17	1506.110458
12	Carveol	99-48-9	C10H16O	23.76	1333.790581
13	Verbenone	1196-01-6	C10H14O	24.07	1355.887054
14	Copaene	3856-25-5	C15H24	25.06	1529.875986
15	β-Caryophyllene	87-44-5	C15H24	26.28	1414.421828
16	Humulene	6753-98-6	C15H24	27.2	1490.208783
17	Germacrene D	23986-74-5	C15H24	27.93	1700.91047
18	γ-Elemene	29873-99-2	C15H24	28.34	2361.044177
19	Naphthalene	483-76-1	C15H24	28.98	1523.343284

## Data Availability

The raw data of RNA-seq in *S. tenuifolia* under salt stress were uploaded into the National Genomics Data Center and the accession number was PRJCA026971. The amino acid sequences of StMYB71 and StMYB8774 were deposited into GeneBank under accession numbers of PQ778212 and PQ778213.

## Supplementary Materials

The supporting information can be downloaded at: https://www.mdpi.com/article/10.3390/plants15101469/s1.

## Abbreviations

AP2/ERF	APETALA2/Ethylene-Responsive Factor
bHLH	basic helix–loop–helix
bZIP	basic leucine zipper
CAT	catalase
DEG	differentially expressed gene
EMSA	electrophoretic mobility shift assay
FDR	false discovery rate
FPKM	fragments per kilobase of transcript per million mapped reads
GC	gas chromatography
GC-MS	gas chromatography–mass spectrometry
GO	gene Ontology
GST	glutathione S-transferase
KEGG	Kyoto Encyclopedia of Genes and Genomes
L3OH	limonene-3-hydroxylase
LC-MS/MS	liquid chromatography–tandem mass spectrometry
MDA	malondialdehyde
MES	2-(N-morpholino)ethanesulfonic acid
MYB	myeloblastosis transcription factor
POD	peroxidase
qRT-PCR	quantitative real-time polymerase chain reaction
SOD	superoxide dismutase
Y1H	yeast one-hybrid
VIGS	directory of open access journals
WRKY	WRKY transcription factor
