# Supplementary material for: MYB Transcription Factors Negatively Regulate StL3OH in Salt Stress Response of Schizonepeta tenuifolia
Source: Plants (Basel). 2026 May 12;15(10):1469. doi: 10.3390/plants15101469 (PMC13210802; doi:10.3390/plants15101469)
Supplement: Supplementary file 1 [file plants-15-01469-s001.zip › plants-4288059-Supplementary materials0430.pdf]

MYB Transcription Factors Negatively Regulate *StL3OH* in Salt Stress Response of *Schizonepeta tenuifolia*

Jingjie Dang [1,2,3], Maoqi Pan [1,2,3], Mengru Sang [1,2,3], Dishuai Li [1,2,3], Mingqiu Shan [1,2,3], Chanchan Liu [1,2,3,*], Qinan Wu [1,2,3,*]

1 National Key Laboratory on Technologies for Chinese Medicine Pharmaceutical Process Control and Intelligent Manufacture, Nanjing University of Chinese Medicine, Nanjing 210023, China; jingjie.dang@njucm.edu.cn (J.D.); pan12@njucm.edu.cn (M.P.); sang7923@163.com (M.S.); 20210651@njucm.edu.cn (D.L.); liuchanchan@njucm.edu.cn (C.L.); wuqn@njucm.edu.cn (Q.W.)

2 Jiangsu Collaborative Innovation Center of Chinese Medicinal Resources Industrialization, Nanjing, 210023, China

3 College of Pharmacy, Nanjing University of Chinese Medicine, Nanjing, 210023, China

* Correspondence: liuchanchan@njucm.edu.cn (C.L.); wuqn@njucm.edu.cn (Q.W.)

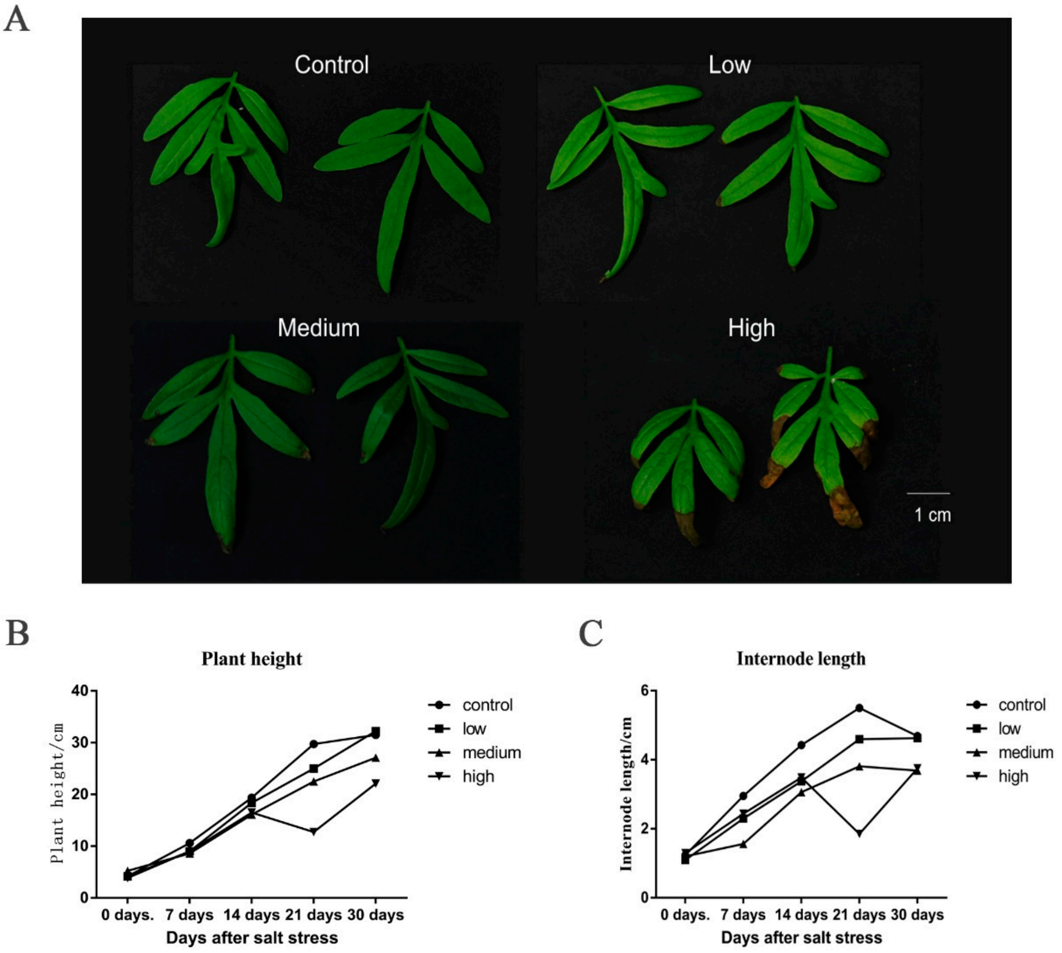

Figure S1 The growth condition of *S. tenuifolia* during the stress process

A, The leaves of *S. tenuifolia* after salt stress; B, The plant height of of *S. tenuifolia* during salt stress; C, The internode length of *S. tenuifolia* during salt stress

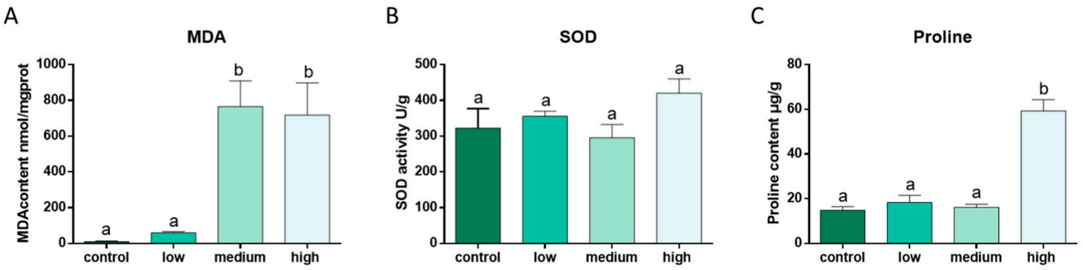

Figure S2 The MDA content (A), SOD activity(B), and the proline content(C) of *S. tenuifolia* after salts stress. Different lowercase letters showed statistically significant at $p < 0.05$.

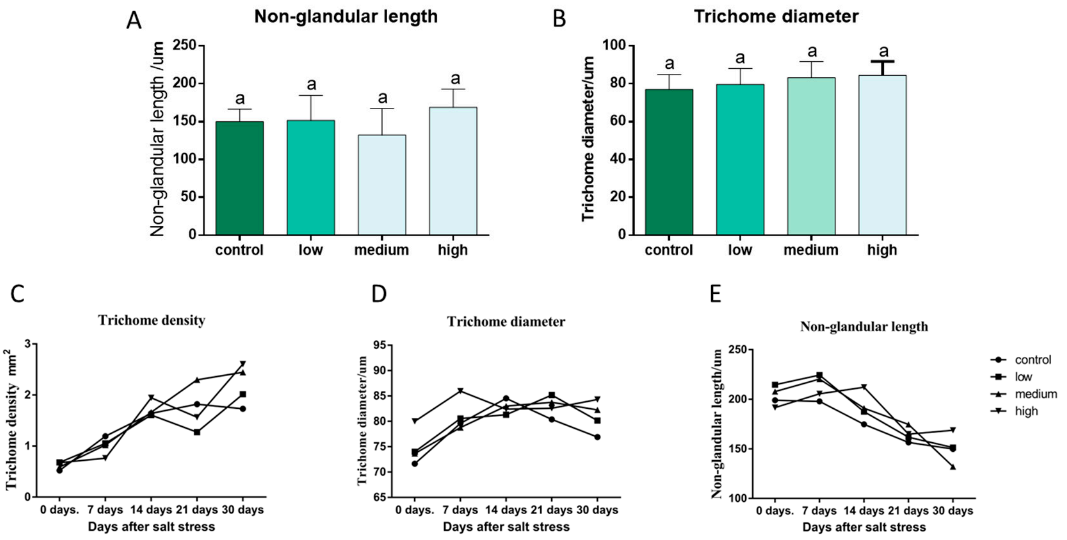

Figure S3 The phenotypic change of *S. tenuifolia* under salt stress

A, The trichome diameter of *S. tenuifolia* after salt stress; B, The non-glandular length of *S. tenuifolia* under salt stress; C, The change of trichome density from *S. tenuifolia* during the stress; D The change of trichome diameter from *S. tenuifolia* during the stress; E The change of non-glandular length from *S. tenuifolia* during the stress. Different lowercase letters showed statistically significant at $p < 0.05$.

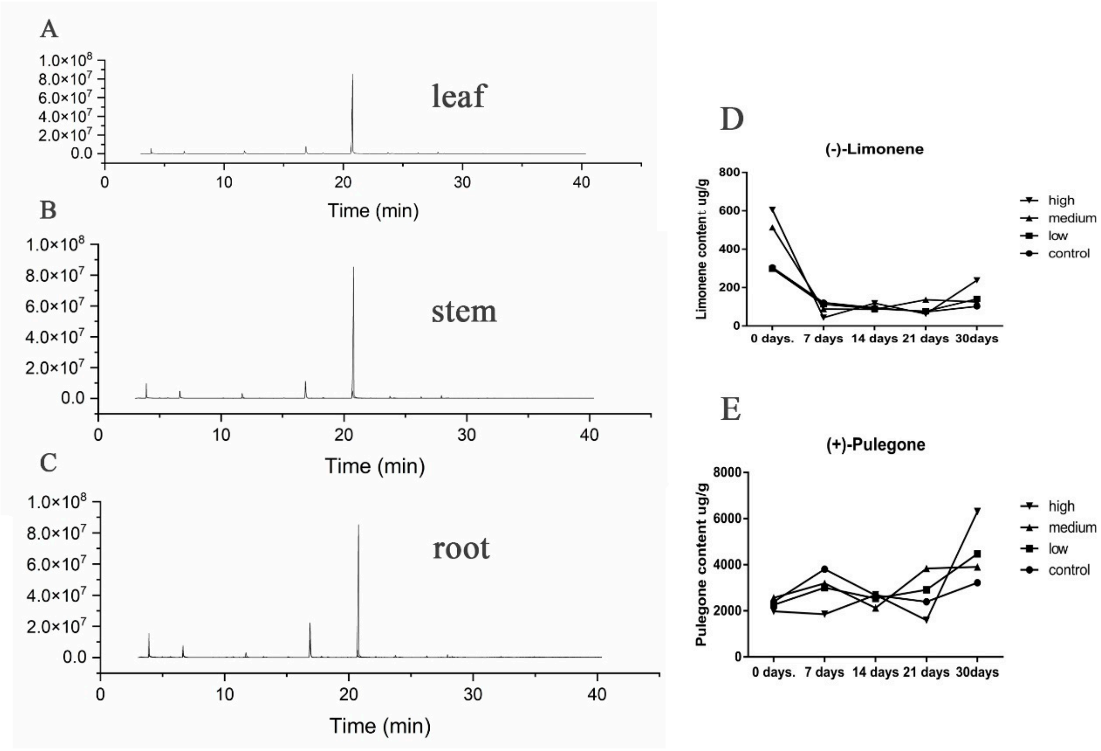

Figure S4 The GC-MS analysis of essential oil from *S. tenuifolia* under salt stress.
The TIC of essential oil from leaves(A), stem(B), and root(C) of *S. tenuifolia* after stress. The content of (-)-limonene(D) and (+)-pulegone of leaves from *S. tenuifolia* during the stress.

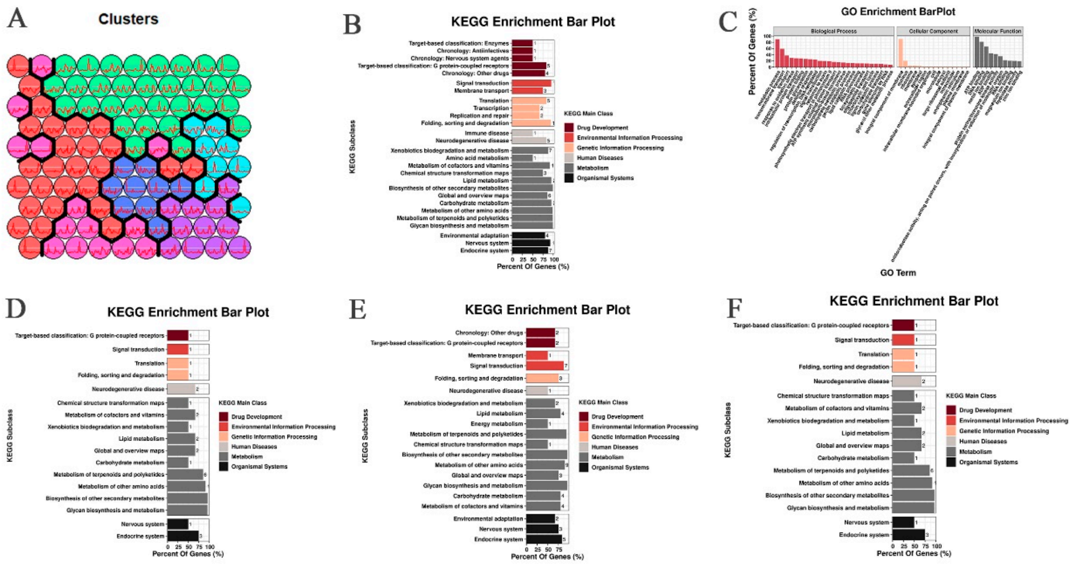

Figure S5 The analysis of transcriptome data

A, SOM analysis of transcriptome data of Schizonepeta tenuifolia; B, The KEGG enrichment analysis of growth-DEGs; C, The GO enrichment analysis of growth-DEGs; D, The KEGG enrichment of DEGs in leaves of Schizonepeta tenuifolia under salt stress; E, The KEGG enrichment of DEGs in stems of Schizonepeta tenuifolia under salt stress; F, The KEGG enrichment of DEGs in roots of Schizonepeta tenuifolia under salt stress

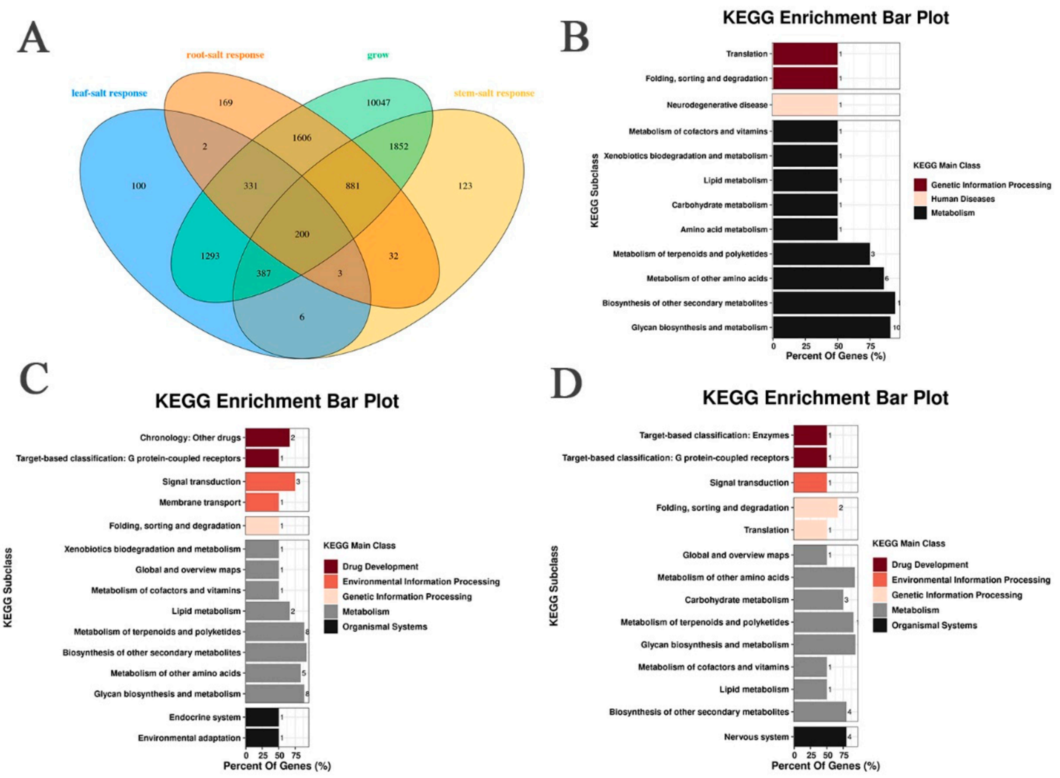

Figure S6 A, The Venn diagram of salt response DEGs and grow DEGs; B, The KEGG enrichment analysis of leaf-salt response DEGs; C, The KEGG enrichment analysis of stem-salt response DEGs; D, The KEGG enrichment analysis of root-salt response DEGs.

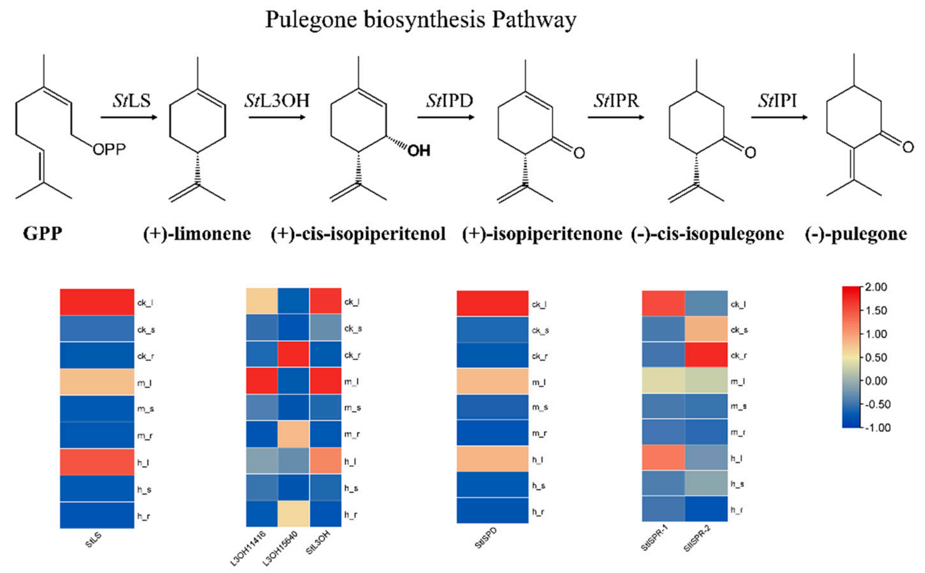

Figure S7 The expression pattern of genes involved in the monoterpenoids biosynthesis pathway after salt stress

Figure S8 The basic and structure analysis of MYB family members in *S. tenuifolia*

A, The gene structure of MYB family members in *S. tenuifolia*; B, The motif analysis of MYB family members in *S. tenuifolia*; C, The conversed domain analysis of MYB family members in *S. tenuifolia*; D, The chromosomal locations of MYB family memebers in *S. tenuifolia*

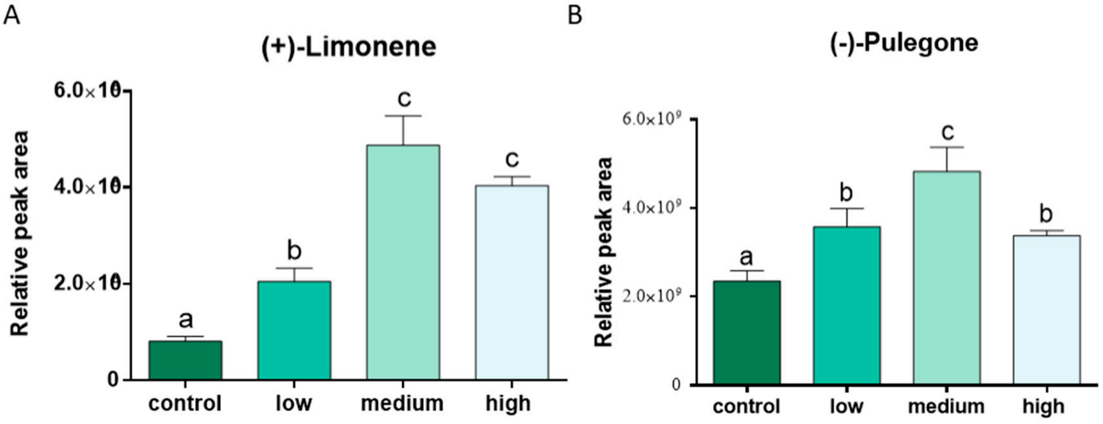

Figure S9 The relative content of (+)-limonene(A) and (-)-pulegone from dried leaves of *S. tenuifolia* after stress. Different lowercase letters showed statistically significant at $p < 0.05$.

Table S1 Primer used for Quantative real-time PCR

Gene ID	Primer Sequence: 5'-3'
StL3OH	F: CTCGCCAATGTAGAGGTT<br>R: GGGAGTGGGGATTAAGAC
StMYB71	F: ACAATACATTCAGGCTCACG<br>R: GTGGGTGTTCCAGTAGTTC
StMYB8774	F: AGATGTGGGAAGAGTTGC<br>R: CTGAGGCGAGCCGAGTA

Table S2 Primers used for Y1H assays

Gene ID	Primer Sequence: 5’-3’
StMYB71-AD	F: gccatggaggccagtgaattcATGGGAAGATCACCTTGCTGTG<br>R: cagctcgagctcgatggatccTTACATGAAATCGTCAAAATCCAAA
StMYB8774-AD	F: gccatggaggccagtgaattcATGGGAAGAACACCGTGCTG<br>R: cagctcgagctcgatggatccTTACATGAAATAATCTAATTCTAAACTCTCCG
StL3OHpro-1-NC-AbAi	F: agtggtctctgtccagtcctGCCATGTACGATGTGTGCTGTC<br>R: ggtctcagcagaccacaagtGTATGATACAAATTTGAAATTTTGGCG
StL3OHpro-2-NC-AbAi	F: agtggtctctgtccagtcctATGAAGATTAGTTTTCATGTATGATGCA<br>R: ggtctcagcagaccacaagtCTCAATTAATCCTCCTAATTAATCAACTAA

Table S3 Primers used for EMSA

Gene ID	Primer Sequence: 5'-3'
StMYB71-GST	F: ttccaggggcccctgggatccATGGGAAGATCACCTTGCTGTG<br>R: ctcgagtcgacccgggaattcTTACATGAAATCGTCAAAATCCAAA
StMYB8774-GST	F: ttccaggggcccctgggatccATGGGAAGAACACCGTGCTG<br>R: ctcgagtcgacccgggaattcTTACATGAAATAATCTAATTCTAAACTCTCCG
L3OHprobe	F: Biotin-tgagagtactaagtagtactctatgtgtcccgttgttattggcctgtattctttttgagttgtctcatt<br>R: aatgagacaactcaaaaaagaatacaggccaataacaacgggacacatagagtactacttagtactctca
L3OHprobe-Competitor	F: tgagagtactaagtagtactctatgtgtcccgttgttattggcctgtattctttttgagttgtctcatt<br>R: aatgagacaactcaaaaaagaatacaggccaataacaacgggacacatagagtactacttagtactctca

Table S4: Primer used for construction of vector for VIGS

Gene ID	Primer Sequence: 5’-3’
StMYB71-TRV2	F: cgtgagctcggtaccggatccGAGCGCCGAAGCCGAGTT<br>R: gtgagtaaggttaccgaattcTCACCAAATATCAACAACAATTCTTTC
StMYB8774-TRV2	F: cgtgagctcggtaccggatccTTACATGAAATAATCTAATTCTAAACTCTCCG<br>R: gtgagtaaggttaccgaattcCTTACAGATGCTTCACCTCAAAATCA

Table S5 Summary of Illumina sequencing data

Sample[a]	Raw Data Reads(Gb)	Valid Data Reads(Gb)	Valid Data Q20(%)[b]	Valid Data Q30(%)	Valid Data Ratio(%)
ck_l_1	6.34	6.26	99.98	97.08	98.76
ck_l_2	6.75	6.67	99.98	96.96	98.77
ck_l_3	6.22	6.15	99.98	97.01	98.79
ck_s_1	6.91	6.81	99.98	97.06	98.57
ck_s_2	6.72	6.64	99.98	96.94	98.82
ck_s_3	6.58	6.50	99.98	97.12	98.79
ck_r_1	6.42	6.31	99.98	96.76	98.24
ck_r_2	6.67	6.59	99.98	96.89	98.76
ck_r_3	7.03	6.93	99.98	96.57	98.67
m_l_1	7.67	7.57	99.98	96.82	98.67
m_l_2	7.05	6.94	99.98	96.98	98.53
m_l_3	7.27	7.18	99.98	96.98	98.72
m_s_1	7.05	6.90	99.98	96.80	98.65
m_s_2	7.20	7.10	99.98	96.94	98.57
m_s_3	7.75	7.65	99.98	96.72	98.72
m_r_1	6.48	6.40	99.98	96.53	98.71
m_r_2	7.36	7.35	99.98	96.70	98.52
m_r_3	6.41	6.42	99.98	96.51	98.49
h_l_1	6.21	6.12	99.98	96.58	98.60
h_l_2	6.31	6.23	99.98	96.48	98.71
h_l_3	6.33	6.24	99.98	96.49	98.69
h_s_1	6.70	6.61	99.98	96.65	98.68
h_s_2	6.73	6.63	99.97	96.56	98.55
h_s_3	5.00	4.94	99.90	93.91	98.83
h_r_1	6.37	6.28	99.96	94.72	98.53
h_r_2	7.54	7.44	99.98	96.39	98.62
h_r_3	6.49	6.41	99.98	96.46	98.74

[a] The ck represent libraries constructed by control group of samples, m represents the libraries constructed

by medium groups of samples under salt stress, and h means libraries constructed by high group of samples under salt stress. Number indicates three biological replicates; [b] Q20 percentage represents percentage of bases with a Phred value >20.

Table S6 Summary of StMYB family memebers

ID	MW	pI	ORF length	amino acid length	Intron number	Exon number
Sch000025404	17526.7	3.72	468	156	0	1
Sch000016947	34415.19	4.59	921	307	2	3
Sch000002866	20058.92	4.73	537	179	1	2
Sch000021292	17789.83	4.78	474	158	0	1
Sch000000660	23213.48	4.84	609	203	2	3
Sch000014223	29827.64	4.87	825	275	0	1
Sch000004041	35265.17	4.87	945	315	2	3
Sch000003511	37875.95	4.98	1017	339	2	3
Sch000001594	29241.37	5.06	771	257	2	3
Sch000004436	33112.11	5.15	885	295	2	3
Sch000007178	39743.17	5.17	1065	355	2	3
Sch000021160	27964.35	5.18	729	243	2	3
Sch000005920	34594.85	5.19	924	308	2	3
Sch000018131	37456.26	5.22	996	332	2	3
Sch000007376	107233.28	5.25	2859	953	3	4
Sch000002260	57986.59	5.3	1554	518	11	12
Sch000016989	42505.84	5.35	1185	395	2	3
Sch000026656	70848.47	5.35	1947	649	6	7
Sch000026698	104589	5.36	2817	939	8	9
Sch000027616	37985.19	5.38	1020	340	2	3
Sch000011575	19457.94	5.4	510	170	1	2
Sch000008546	34500.24	5.4	924	308	2	3
Sch000010462	56341.7	5.47	1539	513	2	3
Sch000004705	26787.92	5.48	699	233	2	3
Sch000013115	26616.82	5.56	711	237	2	3
Sch000018104	34203.59	5.57	909	303	2	3
Sch000002874	30793.26	5.59	810	270	2	3

ID	MW	pI	ORF length	amino acid length	Intron number	Exon number
Sch000029464	30793.26	5.59	810	270	2	3
Sch000001250	39181.23	5.65	1020	340	2	3
Sch000020922	34150.5	5.67	894	298	2	3
Sch000028495	35350.48	5.69	960	320	2	3
Sch000028568	31807.6	5.73	867	289	0	1
Sch000012248	56699.78	5.76	1563	521	2	3
Sch000019203	28730.5	5.78	750	250	2	3
Sch000025545	51137.46	5.78	1401	467	2	3
Sch000013184	35954.02	5.8	966	322	2	3
Sch000009295	61893.74	5.8	1614	538	1	2
Sch000025134	36213.3	5.81	951	317	2	3
Sch000000071	37159.56	5.85	996	332	2	3
Sch000030250	17166.42	5.86	450	150	2	3
Sch000009583	37483.91	5.86	987	329	2	3
Sch000014083	68650.14	5.89	1887	629	5	6
Sch000026240	33047.29	5.93	900	300	2	3
Sch000019464	51991.41	5.93	1377	459	11	12
Sch000001690	47362.62	5.97	1296	432	2	3
Sch000008586	32912.06	5.98	891	297	2	3
Sch000003477	34313.76	5.99	897	299	2	3
Sch000012506	35636.71	5.99	930	310	2	3
Sch000003765	29901.38	6	804	268	2	3
Sch000005500	34252.29	6.15	930	310	2	3
Sch000008774	37007.48	6.15	987	329	2	3
Sch000019825	40773.01	6.17	1134	378	3	4
Sch000016115	43773.49	6.19	1152	384	3	4
Sch000001254	28510.37	6.2	747	249	2	3
Sch000011059	30578.26	6.2	834	278	2	3

ID	MW	pI	ORF length	amino acid length	Intron number	Exon number
Sch000019363	31943.33	6.2	861	287	2	3
Sch000011424	33476.48	6.2	882	294	2	3
Sch000016959	26036.98	6.21	711	237	1	2
Sch000003789	36639.96	6.21	978	326	2	3
Sch000017048	36119.95	6.25	993	331	1	2
Sch000020281	33289.55	6.26	882	294	2	3
Sch000000431	30393.91	6.27	804	268	2	3
Sch000026614	21687.35	6.32	579	193	2	3
Sch000020033	34191.01	6.37	927	309	2	3
Sch000022598	35678.84	6.45	945	315	2	3
Sch000006843	30499.17	6.48	786	262	2	3
Sch000002130	36149.03	6.52	972	324	1	2
Sch000018640	24524.4	6.53	636	212	3	4
Sch000017383	34309.29	6.61	921	307	2	3
Sch000025397	44763.72	6.83	1227	409	2	3
Sch000019206	35965.27	6.84	951	317	2	3
Sch000027474	27820.7	6.9	717	239	2	3
Sch000018454	30000.6	6.96	804	268	2	3
Sch000018397	27378.47	7.04	723	241	2	3
Sch000012461	37367.57	7.05	996	332	2	3
Sch000015875	124083.76	7.09	3321	1107	10	11
Sch000001365	41495.41	7.22	1101	367	2	3
Sch000008609	23305.43	7.68	609	203	2	3
Sch000006010	35073.66	7.68	963	321		
Sch000008970	22019	7.71	573	191	2	3
Sch000013493	34351.64	8.03	927	309	2	3
Sch000016262	13132.73	8.04	345	115	1	2
Sch000028427	38545.41	8.1	1026	342	2	3

ID	MW	pI	ORF length	amino acid length	Intron number	Exon number
Sch000018604	37122.38	6.46	1005	335	2	3
Sch000011725	34343.66	6.5	930	310	1	2
Sch000009290	32942.09	6.74	894	298	2	3
Sch000019516	18109.26	6.9	474	158	1	2
Sch000030195	22048.99	7.12	573	191	2	3
Sch000025036	30270.03	7.14	789	263	2	3
Sch000024808	42716.95	8.02	1125	375	2	3
Sch000007365	31415.8	8.19	825	275	2	3
Sch000015101	28699.08	8.2	753	251	2	3
Sch000019608	26154.94	8.27	693	231	2	3
Sch000002066	37658.59	8.31	1020	340	2	3
Sch000015932	35013.32	8.54	951	317	2	3
Sch000020752	27462.83	8.56	714	238	2	3
Sch000005704	36234.92	8.63	963	321	2	3
Sch000027973	8948.1	8.66	246	82	1	2
Sch000029377	37973.37	8.68	1008	336	2	3
Sch000018410	27754.64	8.72	714	238	2	3
Sch000010435	31880.74	8.73	879	293	2	3
Sch000017878	32725.39	8.74	879	293	2	3
Sch000029032	87046.21	8.75	2286	762	4	5
Sch000008787	30056.08	8.82	816	272	2	3
Sch000028205	28146.85	8.84	753	251	1	2
Sch000003290	28847.24	8.85	753	251	2	3
Sch000000089	37589.54	8.86	1023	341	2	3
Sch000003705	30086.63	8.9	795	265	2	3
Sch000020928	33567.81	8.91	897	299	2	3
Sch000005295	35054.88	8.96	921	307	2	3
Sch000010565	93958.62	8.97	2565	855	14	15

ID	MW	pI	ORF length	amino acid length	Intron number	Exon number
Sch000017404	26435.23	8.99	687	229	2	3
Sch000026842	26636.29	9.01	711	237	1	2
Sch000015543	32099.52	9.02	879	293	1	2
Sch000018969	29816.6	9.03	801	267	1	2
Sch000014212	25317.85	9.07	681	227	0	1
Sch000008946	75026.73	9.09	2013	671	9	10
Sch000021958	25326.93	9.24	645	215	2	3
Sch000016802	35250.99	9.24	951	317	2	3
Sch000022798	23446.57	9.27	612	204	2	3
Sch000006559	38936.39	9.31	1026	342	0	1
Sch000017638	27898.45	9.33	726	242	2	3
Sch000017850	39837.63	9.37	1089	363	0	1
Sch000017577	31519.66	9.38	867	289	0	1
Sch000025250	25715.44	9.4	687	229	1	2
Sch000011497	35631.4	9.42	918	306	4	5
Sch000006307	10207.65	9.5	264	88	2	3
Sch000018975	11149.77	9.51	276	92	2	3
Sch000029595	27283.57	9.51	711	237	3	4
Sch000009587	31995.76	9.56	834	278	1	2
Sch000027006	22493.57	9.63	597	199	2	3
Sch000029896	27565.71	9.68	765	255	1	2
Sch000019004	17653.02	9.74	459	153	1	2
Sch000019717	15927.5	10.03	429	143	2	3
