# Supplementary figures and images for: MYB Transcription Factors Negatively Regulate StL3OH in Salt Stress Response of Schizonepeta tenuifolia
Source: Plants (Basel). 2026 May 12;15(10):1469. doi: 10.3390/plants15101469 (PMC13210802; doi:10.3390/plants15101469)

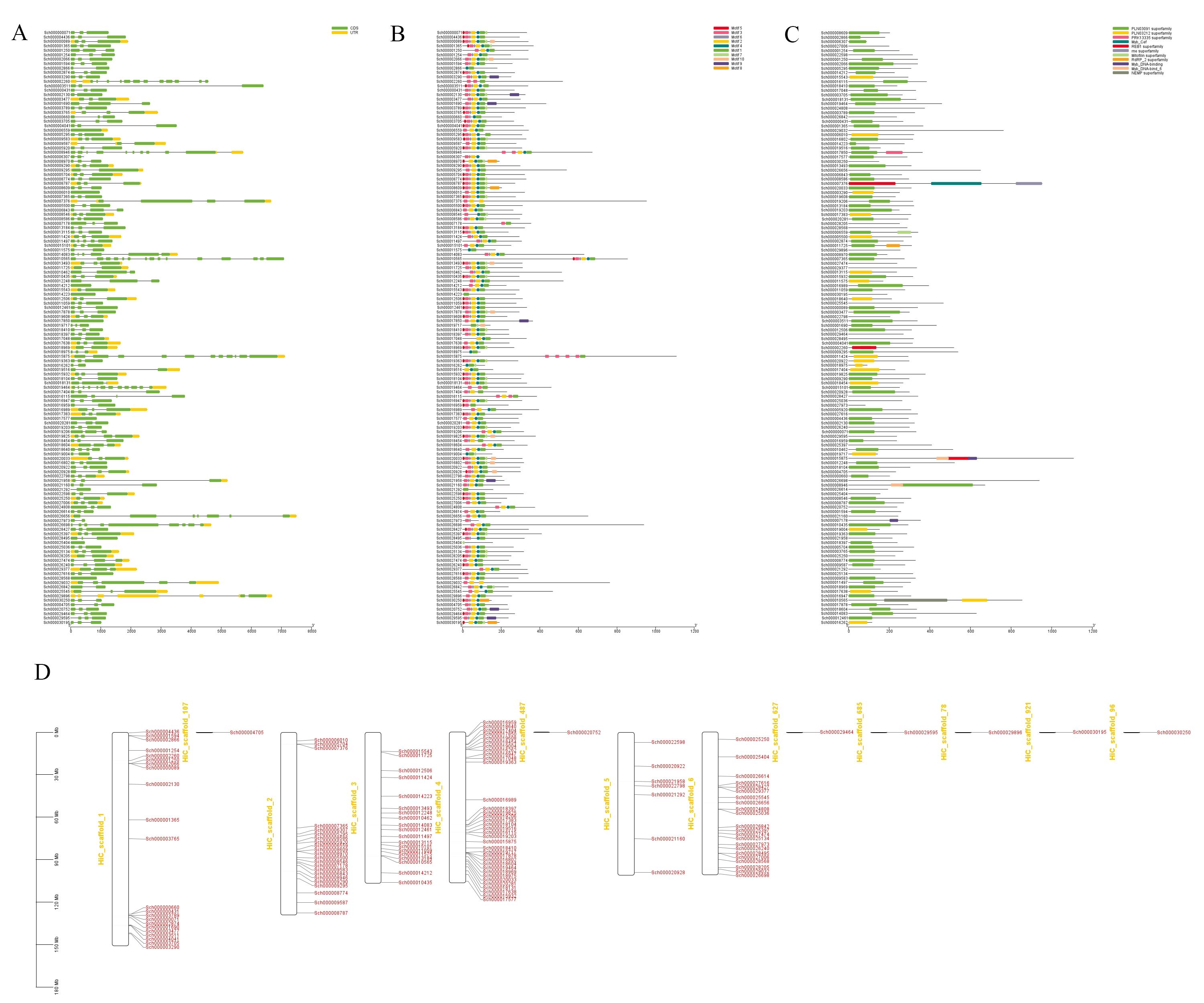

Supplement: Supplementary file 1 [file plants-15-01469-s001.zip › Figure S8.jpg]
